# Enforced Expression of Bcl-2 Selectively Perturbs Negative Selection of Dual Reactive Antibodies

**DOI:** 10.1155/2001/83595

**Published:** 2001

**Authors:** Evangelia Notidis, Shailaja Hande, Tim Manser

**Affiliations:** Department of Microbiology and Immunology The Kimmel Cancer Institute Thomas Jefferson Medical College Philadelphia PA 19107 USA

**Keywords:** Bcl-2, B-lymphocytes, clonal selection, peripheral tolerance, somatic hypermutation

## Abstract

We investigated the role of apoptosis in the development of B cell memory by analyzing the (p-azophenylarsonate) Ars response in a line of A strain mice in which expression of human Bcl-2 was enforced in the B cell compartment. Previous studies of the Ars immune response in these A. Bcl-2 mice, demonstrated that a large percentage of the antibodies expressed by the Ars induced memory B cell compartment had accumulated point mutations via somatic hypermutation that increased their affinity for both Ars and the autoantigen DNA (“dual reactive” antibodies). This was in sharp contrast to normal A strain mice which displayed no dual reactive B cells in their Ars induced memory B cell compartment. These data suggested that interference with apoptotic pathways regulated by Bcl-2 allows developing memory B cells that have acquired autoreactivity to bypass a peripheral tolerance checkpoint. Further studies of these mice, reported here, demonstrate that enforced expression of Bcl-2 does not alter serum antibody affinity maturation nor positive selection of B cells expressing somatically mutated antibody with an increased affinity for Ars. Moreover, the somatic hypermutation process was unaffected in A. Bcl-2 mice. Thus, enforced expression of Bcl-2 in A. Bcl-2 mice appears to selectively alter a negative selection process that operates during memory B cell differentiation.


*Developmental Immunology*, 2001, Vol. 8(3-4), pp. 223-234
Reprints available directly from the publisher
Photocopying permitted by license only

© 2001 OPA (Overseas Publishers Association)
N.V. Published by license under
the Harwood Academic Publishers imprint,
part of The Gordon and Breach Publishing Group,
a member of the Taylor and Francis Group

### Enforced Expression of Bcl-2 Selectively Perturbs Negative Selection of Dual Reactive Antibodies

EVANGELIA NOTIDIS, SHAILAJA HANDE and TIM MANSER*

*Department of Microbiology and Immunology and The Kimmel Cancer Institute Thomas Jefferson Medical College Philadelphia PA 19107 U.S.A*

We investigated the role of apoptosis in the development of B cell memory by analyzing the (p-azophenylarsonate) Ars response in a line of A strain mice in which expression of human Bcl-2 was enforced in the B cell compartment. Previous studies of the Ars immune response in these A. Bcl-2 mice, demonstrated that a large percentage of the antibodies expressed by the Ars induced memory B cell compartment had accumulated point mutations via somatic hypermutation that increased their affinity for both Ars and the autoantigen DNA ("dual reactive" antibodies). This was in sharp contrast to normal A strain mice which displayed no dual reactive B cells in their Ars induced memory B cell compartment. These data suggested that interference with apoptotic pathways regulated by Bcl-2 allows developing memory B cells that have acquired autoreactivity to bypass a peripheral tolerance checkpoint. Further studies of these mice, reported here, demonstrate that enforced expression of Bcl-2 does not alter serum antibody affinity maturation nor positive selection of B cells expressing somatically mutated antibody with an increased affinity for Ars. Moreover, the somatic hypermutation process was unaffected in A. Bcl-2 mice. Thus, enforced expression of Bcl-2 in A. Bcl-2 mice appears to selectively alter a negative selection process that operates during memory B cell differentiation.

*Keywords:* Bcl-2, B-lymphocytes, clonal selection, peripheral tolerance, somatic hypermutation

### INTRODUCTION

Upon activation by antigen, mature B-lymphocytes migrate to the primary follicle and establish distinct microenvironments known as germinal centers (GCs). Once located in the GC, B cells undergo a series of differentiation events. One of these events is the activation of a somatic hypermutation mechanism, which rapidly introduces point mutations into Ig variable (V) regions (Tsiagbe et al., 1996). Although not completely random, somatic hypermutation can result in various V region phenotypes. For instance, some mutations may disrupt the structural integrity of the V region leading to a loss of function (Manser et al., 1987a; Schlomchik et al., 1989), whereas others can result in an increase in antibody affinity (Berek et al., 1987; Sharon et al., 1989; Weiss et al., 1990). These high affinity variants provide the substrate for serum antibody affinity maturation. In addition, certain mutations can often fortuitously generate V regions with an autoreactive phenotype (Casson and Manser, 1995; Hande and Manser 1997).

B cells in the GC have also been demonstrated to exhibit a high incidence of apoptosis (MacLennan,

* To whom correspondence and proofs should be addressed. Phone number: (215)-503–4543; fax number: (215)-923–4153.

1994) which is believed to mediate the disappearance of low affinity V region mutants, autoreactive mutants, or both. The susceptibility of GC B cells to apoptosis can be attributed to factors such as the absence of the anti-apoptotic protein Bcl-2 (Martinez-Valdez et al., 1996) and high expression of Fas (CD95) (Tsiagbe et al., 1996). Evidence to support the sensitivity of GC B cells to apoptotic signals has also been demonstrated in studies where GC B cells interact with high levels of cognate soluble antigen *in situ* (Pulendran et al., 1995; Shokat and Goodnow, 1995; Han et al., 1995); a situation argued to mimic a form of tolerance induction *in vivo*.

During the immune response to the hapten Ars in A/J mice, there is the predominance of a particular clonotype termed the canonical clonotype (Manser et al., 1987b; Rathbun et al., 1989; Wysocki et al., 1987). Canonical antibodies are composed of specific heavy and light chain V regions that prior to somatic mutation, differ only at their $V_H$-D and D-$J_H$ segment junctions. Previous *in vitro* mutagenesis studies of canonical monoclonal antibodies (mAbs) have illustrated that certain mutations in the CDRs often result in simultaneous increases in the affinity for foreign (Ars) and self (DNA) antigen (Casson and Manser, 1995; Hande and Manser 1997). However, previous *in vivo* data revealed that such "dual" reactive canonical antibodies are absent from the Ars-induced memory B cell compartment (Naparstek et al., 1986). Suggesting therefore, that dual reactive mutants were absent from the memory compartment of normal healthy individuals due to the action of a peripheral tolerance checkpoint. In order to test this hypothesis we have studied the immune response to Ars in A strain mice which constitutively expressed Bcl-2 in the B cell compartment.

Initial analyses of the Ars response in A.Bcl-2 mice indicated that most aspects of this response were not overtly altered. Participation of canonical anti-Ars clonotypes in the immune response and somatic mutation of canonical antibodies appeared comparable to normal A strain mice (Hande et al., 1998). However, whereas dual reactive hybridomas are not isolated from secondary and tertiary fusions from A strain mice, such hybridomas made up approximately 25% of the canonical expressing secondary and tertiary hybridomas isolated from A.Bcl-2 mice (Hande et al., 1998). These data suggested that altering apoptosis obstructs negative selection of dual reactive clones in the periphery.

B cell tolerance has also been studied in other transgenic mouse lines in which Bcl-2 expression is enforced in the B cell compartment. In particular, a recent study by Kuo et al., (1999) suggests that altering Bcl-2 expression affects central tolerance but does not affect peripheral tolerance. Specifically, dual reactive anti-DNA, anti-phosphorylcholine (PC) B cells could be recovered by hybridoma technology late in the primary response but did not appear in the anti-PC memory B cell compartment (Kuo et al., 1999). Additional studies from the same group also argue that although Bcl-2 does not affect the frequency of somatic mutation, it impairs the clustering of mutations to "hot spot" motifs (Kuo et al., 1997). In addition to Bcl-2 transgenics, maturation of the B cell response has also been investigated in mice over-expressing the anti-apoptotic protein Bcl-$x_L$ in B cells (Fang et al., 1996). Studies of these transgenic mice demonstrated that over-expression of Bcl-$x_L$ alters affinity maturation allowing low affinity B cells to participate in the response (Takahashi et al., 1999).

Given these observations of others, one could argue that if positive selection, somatic mutation, or both are altered by enforced expression of Bcl-2 in A. Bcl-2 mice, then the effects on the frequency of Ars-induced "dual reactive" antibodies could be indirect. For instance, if the pattern of somatic mutation in a V gene was altered by Bcl-2, the frequency of "dual reactive" antibodies encoded by that V gene might change. Alternatively, if enforced expression of Bcl-2 lowered the "stringency" of positive selection, dual reactive B cells, which would be expected to bind less stimulatory foreign antigen due to "receptor blockade" by self antigen(s) might nevertheless enter the memory B cell compartment.

For these reasons, we decided to closely examine positive selection and V region hypermutation in A. Bcl-2 mice undergoing an Ars immune response. We found that canonical antibodies elicited in these mice displayed a normal frequency of V region somatic hypermutation, with point mutations clustered at mutational "hot spots". We also observed an increase in serum antibody affinity, which was comparable to normal controls. In addition to the existence of dual reactive canonical antibodies, the conventional ($Ars^+$ $DNA^-$) canonical antibodies expressed in the memory B cell compartment displayed increased affinity for Ars due to somatic mutation. Therefore, our results suggest that apoptotic pathways regulated by Bcl-2 do not influence the mechanisms or the regulation of somatic hypermutation or positive selection of antibody mutants with increased affinity.

### RESULTS

### Bcl-2 Transgene Expression Is Predominant In The B Cell Compartment of A. Bcl-2 Mice

To obtain A strain mice with constitutive B cell expression of Bcl-2, the M23 Bcl-2 transgenic mouse line (Smith et al., 1994) was backcrossed to the A/J mouse strain for 4–6 generations. The M23 transgene is a reconstruction of the t(14;18)(q32;q21) translocation observed in B cell lymphomas and has a central region of human Bcl-2 cDNA that contains the exon II – exon III juncture (McDonnell et al., 1989). The expression of the human Bcl-2-Ig fusion "minigene" allows the expression of transgene encoded Bcl-2 protein to be unambiguously distinguished from endogenous mouse Bcl-2 expression. To evaluate this expression, immunohistochemical staining was performed on frozen spleen sections from 5-week-old naive A.Bcl-2 mice, using a mouse IgG1 mAb that recognizes human Bcl-2 protein followed by a goat anti-mouse IgG mAb. Transgenic Bcl-2 expression was predominantly localized in follicles, including GCs, with a more scattered expression in the PALS and red pulp (Figure 1A). As expected, transgene negative littermates displayed only occasional $IgG_1$ positive B cells (Figure 1B).

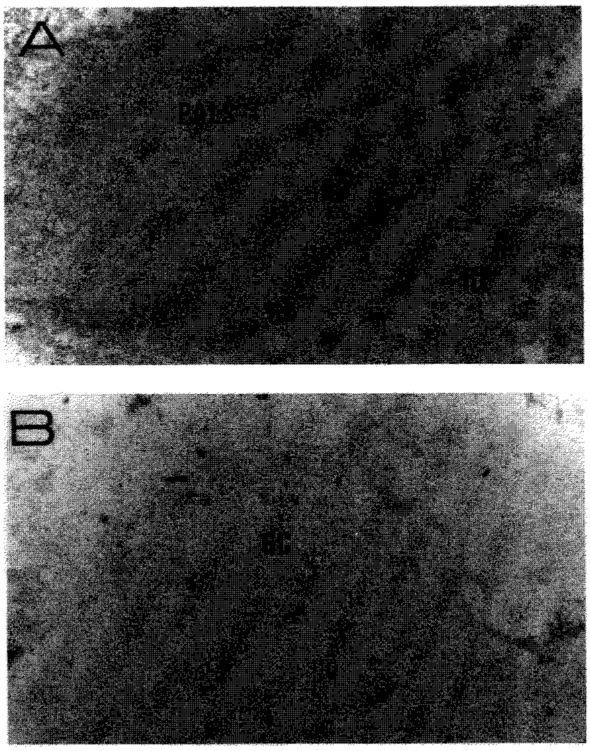

FIGURE 1 Immunohistochemical Analysis Of Transgene Expression In Naive Spleens From A. Bcl-2 Mice. Spleen sections of a 5 week old A.Bcl-2 mouse (A) and a 5 week old control littermate (B) were stained for the expression of human Bcl-2 protein (stained blue) and germinal centers (GCs) (stained red). A.Bcl-2 mice heavily expressed the human Bcl-2 protein in their follicles and GCs, and exhibited sporadic expression in other regions of the spleen, such as the red pulp. As expected, control littermates exhibited no expression of the transgene and only show sporadic presence of IgGl positive cells

### Levels Of Serum Autoantibodies In A. Bcl-2 Mice Are Comparable To Control Littermates

In our initial study, we used a hybridoma approach to evaluate how positive and negative selection events may be altered during the maturation of the anti-Ars response in A. Bcl-2 mice (Hande et al., 1998). The vast majority of the canonical hybridomas isolated expressed mAbs that were class-switched, had undergone extensive somatic mutation and were Ars binding. In addition, data from this analysis demonstrated that altering apoptotic pathways regulated by Bcl-2 allowed for the entry of dual reactive B cells into the memory compartment. Sequence analysis of the Ars-DNA binding V regions that were isolated revealed that the overall pattern of somatic mutation did not differ from that observed in the V regions isolated from the memory compartment of normal A strain mice (Manser, 1991). Several of these dual reactive mAbs also displayed relative DNA binding that was as good as or better than the autoantibody 3H9 which was isolated from a diseased MRL lpr/lpr mouse (Shlomchik et al., 1987). Thus, enforced Bcl-2 expression in the B cell compartment allowed for the expansion of canonical dual reactive B cells. Consequently, we decided to investigate whether these B cells produced any serum antibody.

We primed both A.Bcl-2 mice and transgene negative control littermates with Ars-KLH in CFA. The mice were then bled, boosted with Ars-KLH in PBS and bled again following secondary and tertiary immunizations. Bulk anti-ssDNA, anti-dsDNA, and anti-Ars antibody titers were then determined and compared between A.Bcl-2 mice (Figure 2A-2C) and control littermates (Figure 2D-2F). As is shown in Figure 2A-2F and in our previous analysis (Hande et al., 1998), the serum antibody levels in both groups are comparable, with a slight increase in the level of anti-ssDNA and anti-dsDNA antibodies in the A. Bcl-2 mice during tertiary immune responses (Figure 2C). This increase may be attributed to the overall increase in serum Ig, which is observed in these mice during a tertiary immune response (Figure 3B). This is in accord with previous studies of Bcl-2 transgenic mice, which demonstrated that they have a prolonged AFC response (Smith et al., 1994) and that they display mild hypergammaglobulinemia with age (McDonnell et al., 1989). Previous studies on autoreactive Ig transgenic mouse models have demonstrated that although B cells with a low affinity for DNA persist in the periphery and can be rescued as hybridomas, they do not produce serum antibody (Erikson et al., 1991). Hence, although our previous studies showed that Ars-DNA dual reactive antibodies could be isolated at the level of hybridomas, they could not be detected in the serum after Ars immunization, suggesting that dual reactive B cells, like anti-DNA Ig transgenic B cells, are regulated such that they do not secrete antibody.

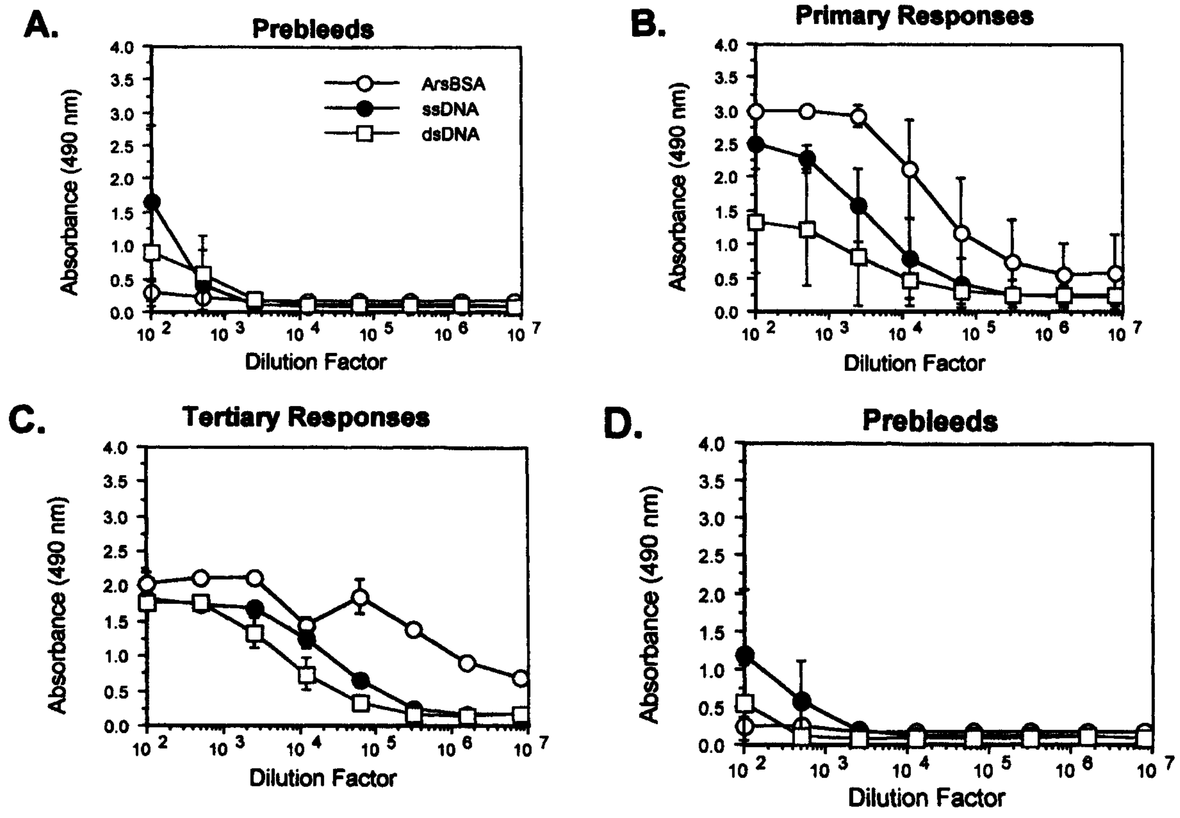

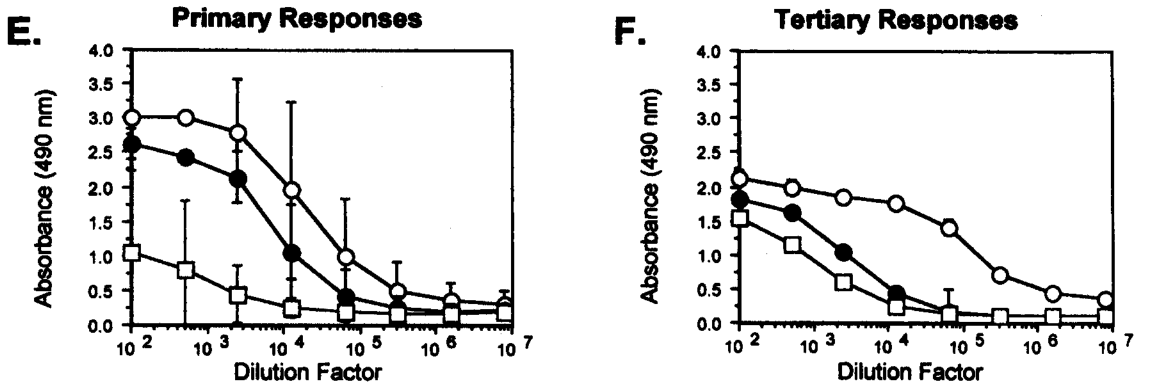

FIGURE 2 Binding Activities Of Antibodies In Sera Of A.Bcl-2 Mice And Control Littermates Following Immunization With Ars-KLH Pooled sera from four to eight A.Bcl-2 mice (A-C) and four to five control littermates (D-F) were analyzed for anti-Ars, anti-ss and anti-dsDNA IgG titers prior to immunization, and after primary (day 8) and tertiary (day 7) immunizations with Ars-KLH. Antibody titers were similar between the two groups prior to immunization and after primary immunization, whereas A. Bcl-2 mice expressed a slight increase over their control littermates after the tertiary immunization

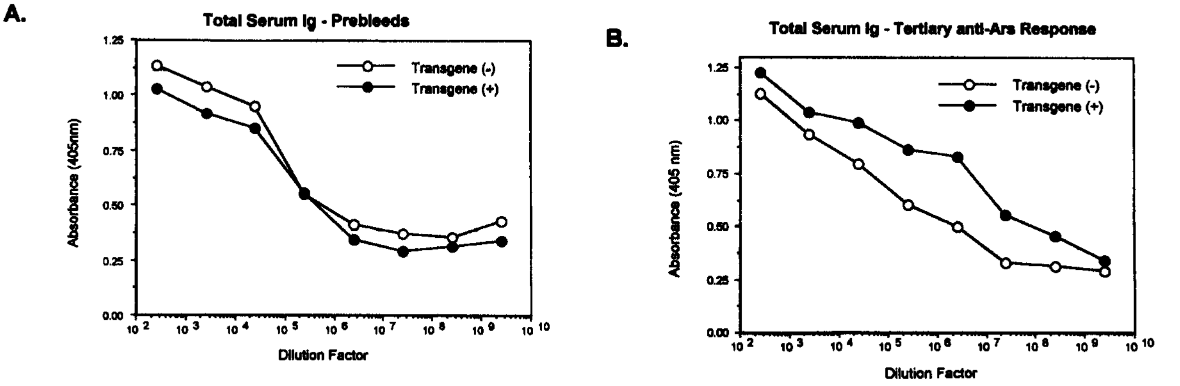

FIGURE 3 Total Serum Immunoglobulin In A. Bcl-2 Mice And Control Littermates. Total serum immunoglobulin was compared between A.Bcl-2 mice and control littermates prior to Ars-KLH immunization at 8 weeks old (A) and after tertiary Ars-KLH immunization at 6 months of age (B). Levels of total serum Ig antibody was comparable in both groups prior to immunization, whereas A. Bcl-2 mice express slightly elevated levels of serum Ig following a tertiary response. Increased levels of total serum Ig can be attributed to the incidence of the mild hypergammaglobulinemia that is associated with age in M23 transgenic mice (McDonnell et al., 1989)

### Serum Antibody In A. Bcl-2 Mice Shows Evidence Of Affinity Maturation

In order to examine antibody affinity maturation in A. Bcl-2 mice, sera from A.Bcl-2 mice undergoing immune responses to Ars-KLH were analyzed by an altered ligand density ELISA. High affinity serum antibody will be distinguished from low affinity serum antibody since high affinity serum antibody will bind equally well to both hapten densities. Conversely, low affinity antibody will bind densely conjugated protein much better than sparsely conjugated protein. The relative affinities of the serum antibodies were compared to the affinities of 36–65, an unmutated canonical mAb and to 36–71, a canonical mAb which possesses mutations that increase its affinity for Ars 100-fold (Vora and Manser, 1995).

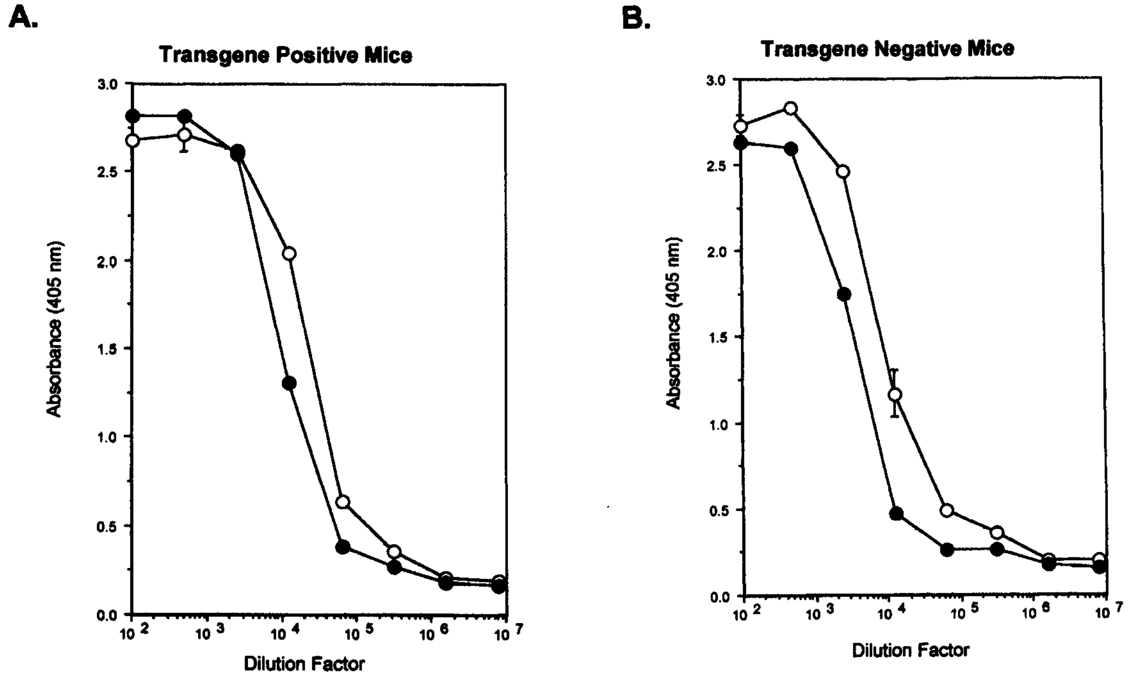

FIGURE 4 Altered Ligand Density ELISA To Measure Serum Antibody Affinity Maturation. Affinity maturation of Ars specific serum antibody of pooled A.Bcl-2 and control serum following secondary immunization with Ars-KLH was determined by altered ligand density ELISA. Pooled sera from A.Bcl-2 mice (A) and strain A transgene negative mice (B) following a secondary immunization with Ars-KLH was tested on a densely conjugated (open circles) and a sparsely conjugated (closed circles) preparation of BSA. The ratio of the 50% binding points of the two different curves for each group was 0.4 for A.Bcl-2 mice and 0.32 for transgene negative control mice. These curves were compared to the binding ratios of 36–65 (Ka = $3 \times 10^5$) which was approximately 0.03 and 36–71 (Ka = $2 \times 10^7$) which was 1

Figure 4 panels A and B demonstrate evidence of normal affinity maturation in the serum antibody of A.Bcl-2 mice. The relative position of the Ars binding curves obtained using either low or high Ars protein conjugates were similar in the case of secondary sera obtained from A.Bcl-2 transgenic mice and strain A transgene negative controls. The near superimposition of the binding curves obtained for the low and high Ars protein conjugates indicate a predominant presence of high affinity Ars antibody in these sera (see legend for details).

### Evidence For Normal Positive Selection Of B Cells With Increased Affinity In A. Bcl-2 Mice

In order to determine whether Ars-driven positive selection was altered in A.Bcl-2 mice, we decided to examine memory B cells expressing canonical antibodies which did not display an affinity for DNA, as determined by ELISA (data not shown). Using the hybridoma approach, we isolated 15 such canonical mAbs from two different A.Bcl-2 mice undergoing a secondary Ars response. All 15 hybridomas expressed IgG antibodies.

A sample of the non-DNA binding canonical mAbs isolated was then chosen for $V_H$ gene sequence analysis. As indicated in Figure 5, all of the hybridomas analyzed showed evidence of somatic mutation in their $V_H$ genes. One of the hybridomas, 10–5-A9, also possesses affinity-enhancing mutations at positions 58 and 59 in CDR2. Specifically, the $V_H$ gene it expresses contains the Thr58- Ile and Lys59- Thr mutations that have previously been shown to result in an increase in affinity for Ars (Sharon et al., 1989).

```

               1                                  10                                      20

CANONICAL     GAG GTT CAG CTT CAG CAG TCT GGA GCT GAG CTG GTG AGG GCT GGG TCC TCA GTG AAG ATG TCC TGC

10-5-A9       --- --- --- --- --- --- --- --- --- --- --- --- --- --- --- --- --- --- --- --- --- ---

10-3-H8       --- --- --- --- --- --- --- --- --- --- --- --- --- --- --- --- --- --- --- --- --- ---

10-8-A10      --- --- --- --- --- --- --- --- --- --- --- --- --- --- --- --- --- --- --- --- --- ---

9-1-A1        --- --- --- --- --- --- --- --- --- --- --- --- --- --- --- --- --- --- --- --- --- --G

                                          30      CDR1                            40

              AAG GCT TCT GGA TAT ACA TTC ACA AGC TAC GGT ATA AAC TGG GTG AAA CAG AGG CCT GGA CAG GGC CTG

10-5-A9       --- --- --- --- --- --- --- --- --- --- --- --- --- --- --- --G --- --- --- --- --- --- ---

10-3-H8       --- --- --- --- --- --- --- --- --T --- --- --- --- --- --- --- --- --- --- --- --- --- ---

10-8-A10      --- --- --- --- --- --- --- --- --- --- --- --- --- --- --- --C G-- --- --- --- --- --- ---

9-1-A1        --- --- --- --- --- --- --- --- -A- -C- --- --- --- --- --- --- --- --- --- --- --- --- ---

                              50              CDR2            58  59              CDR2

              GAA TGG ATT GGA TAT ATT AAT CCT GGA AAT GGT TAT ACT AAG TAC AAT GAG AAG TTC AAG GGC AAG ACC

10-5-A9       --- --- --- --- --- --- --- --- --- --- --- --- -T- C-T --- --- --- --- --- --- --- --- ---

10-3-H8       --- --- --- --- --- --- --- --- --- --A --- --- GT- --A --- --- --- --- --- --- --- --- ---

10-8-A10      --- --- --- --- --- --- --- --- --- --- -A- --- --- --- --- --- --- --- --- --- --- --- ---

9-1-A1        --- --- --- --- --- --- C-- --- --- --- --- --- --- --- -CG --- --- --- --- --- --- --- ---

                  70                                      80                                      90

              ACA CTG ACT GTA GAC AAA TCC TCC AGC ACA GCC TAC ATG CAG CTC AGA AGC CTG ACA TCT GAG GAC TCT

10-5-A9       --- --- --- --- --- --- --- --- --- --- --- --- --- --- --- --- --- --- --- --- --- --- ---

10-3-H8       --- --- --- --- --- --- --- --- --- --- --- --- --A --- --- --- --- --- --- --- --- --- ---

10-8-A10      --- --- --- --- --- --- --- --- T-- --- --- --- --- --C --- --- -A- --- --- --- --- --- ---

9-1-A1        --- --- --- --- --- --- --- --- --- --- --- --- --- --- --- --- --- --- --- --- -G- --- ---

                                          98                      D                       110     JH2

              GCA GTC TAT TTC TGT GCA AGA TCX XXX TAC TAT GGT GGT AGT TAC XXX TTT GAC TAC TGG GGC CAA GGC

10-5-A9       --- --- G-- --- --- --- --- --C AGT --- --- --- --- --- --- TCC --- --- --- --- --- --- ---

10-3-H8       --- --- --- --- --- --- --- --T GTT --- --- --G --- --- --T TCC --- --- --- --- --- --- ---

10-8-A10      --- --- -G- --- --- --- --- --C GGT --- --- --- --- --C --- TCC --- --- --- --- --- --- ---

9-1-A1        --- C-- --- --G --- --- --- --G AAT --- --- --- --- --C --- TCC --- --- --- --- --- --- ---

              ACC ACT CTC ACA GTC TCC TCA

10-5-A9       --- --- --- --- --- --- ---

10-3-H8       --- --- --- --- --- --- ---

10-8-A10      --- --- --- --- --- --- ---

9-1-A1        --- --- --- --- --- --- ---

```

FIGURE 5 Sequences Of The $V_H$ Regions Of $Ars^+$ $DNA^-$ Canonical mAbs. These sequences were determined as described in Materials and Methods and are shown as compared to the unmutated canonical consensus $V_H$ gene sequence. The nucleotides that vary among canonical antibodies at the V-D and D-J junctions are indicated with Xs. Sequence identity is indicated by a dash and differences are shown explicitly. The $V_H$ gene expressed by hybridoma 10–5-A9 also contains the affinity enhancing mutations at positions 58 and 59

TABLE I $K_a$ Values Obtained For $Ars^+$ $DNA^-$ Hybridomas By Fluorescence Quenching Analysis

| *Mouse* | *mAb Name* | *$K_a$ for Ars-Tyr($M^{-1}$)* |
|---|---|---|
| | Unmutated canonical (36–65) | $3.7 \times 10^5$ |
| 5Bcl-9 | 9–1-A1 | $1.6 \times 10^6$ |
| | 9–2-B4 | $8.0 \times 10^6$ |
| 5Bcl-10 | 10–5-C1 | $1.6 \times 10^6$ |
| | 10–3-H8 | $1.7 \times 10^6$ |
| | 10–8-A6 | $1.5 \times 10^6$ |

To examine directly whether there was Ars mediated positive selection of canonical expressing B cells in A. Bcl-2 mice, another sample of the canonical $Ars^+DNA^-$ hybridomas were selected for fluorescence quenching analysis of their expressed mAbs (Table I). As shown in Table I, all of the $K_a$ values obtained for these mAbs were greater than $10^6$ $M^{-1}$. The average affinity for these antibodies was $2.9 \times 10^6$ $M^{-1}$, which is approximately a 10-fold increase over the affinity characteristic of unmutated canonical V regions, such as the monoclonal antibody, 36–65. Such an increase in affinity is characteristic of affinity matured, canonical V regions isolated from Ars-immunized A/J mice (Manser, 1991).

### Enforced Bcl-2 Expression Does Not Affect Clustering Of Mutations In Mutational "Hot Spots"

Somatic hypermutation of Ig genes is not a completely random process. Rather, numerous studies have demonstrated that somatic mutation targets particular "hot spot" motifs (Smith et al., 1996; Rogozin and Kolchanov 1992; Betz et al., 1993; Shapiro et al., 1999). Other studies on Bcl-2 transgenic mice have suggested that although the frequency of V region somatic mutation was normal, the clustering of mutations to "hot spot" motifs was impaired (Kuo et al., 1997). Therefore we decided to compare the mutational frequency and the clustering of mutations to mutational "hot spots" of normal A strain mice to A. Bcl-2 transgenic mice. The sequences chosen for this analysis were from a collection of secondary and tertiary canonical expressing anti-Ars hybridomas.

Recent reports have identified a number of tri- and tetra nucleotide consensus sequences that appear to be targeted for mutation (Smith et al., 1996). We examined the mutations clustered in the tri-nucleotide "hot spots" AGC, TAC, GCT and GTA (Table II) (Smith et al., 1996). In addition, we also examined the level of mutational clustering found at the mutational hot spot designated by the motif purine/G/pyrimidine/A or T or RGYW (Rogozin and Kolchanov 1992) (Table II).

The average percentage of mutations clustered at the tri-nucleotide hot spots AGC/TAC/GCT/GTA for A. Bcl-2 mice was 26%, compared to normal A strain mice which had an average percentage of 22%. Thus, the percentages of mutations clustered at these mutational "hot spot" motifs between the two groups were comparable (Table II). The same conclusion can also be drawn from the comparison made at the mutational hot spot RGYW where the average percent of clustering for the V genes obtained from A.Bcl-2 mice was 37%, compared to controls, which was 32%. The mutational frequencies between the two groups were also comparable, where $V_H$ genes from A/J mice had a mutation frequency of 2.0% and those from A.Bcl-2 mice had a frequency of 1.9%. Therefore, enforced expression of Bcl-2 in A.Bcl-2 mice does not overtly alter the frequency of somatic mutation or "targeting" of these mutations to hot spots.

### DISCUSSION

Preliminary studies using A.Bcl-2 mice indicated that most aspects of the Ars immune response in these mice were comparable to those in non-transgenic A strain mice. Specifically, there was normal participation of the predominant canonical clonotype of the Ars response, comparable levels of anti-Ars serum antibodies, and somatic hypermutation took place in canonical antibody expressing GCs (Hande at al., 1998). Overall, our data were in accord with previous findings, which suggested that many of the antigen-driven stages of T cell dependent B cell differentiation are unaffected in Bcl-2 transgenic mice (Smith et al., 1994).

However, unlike normal A strain mice which lack Ars-DNA dual specific antibody expressing B cells in their memory compartment, such B cells were readily detected in the memory compartment of A.Bcl-2 mice (Hande at al., 1998). These findings stand in contrast to those obtained from studies of the anti-PC response in Eμ*bcl*-2–22 transgenic BALB/c mice. Kuo, et al., (1999) isolated PC-DNA dual reactive B cell hybridomas during the late primary response to PC, but failed to do so in the secondary response. However, unlike the dual reactive hybridomas we had previously isolated, those isolated by Kuo, et al., (1999) were encoded by unmutated $V_H$ genes; suggesting that expression of Bcl-2 in these transgenic mice allows dual reactive B cells to escape central tolerance in the bone marrow. Conversely, our studies indicated that the over-expression of Bcl-2 resulted in dual reactive B cells escaping some form of peripheral tolerance, since all of the hybridomas isolated had evidence of somatic hypermutation (Hande at al., 1998).

TABLE II Clustering Of Mutations To Mutational "Hot Spots"

| | *AGC/TAC/GTA/GCT* | | *purine/G/pyrimidines/A or T (RGYW)* | | | |
|---|---|---|---|---|---|---|
| *Mouse Line* | *Hot Spot Mutations* | *Non-Hot Spot Mutations* | *Hot Spot Mutations* | *Non-Hot Spot Mutations* | *Total Base Pairs*[a] | *Mutation Frequency* |
| CONTROL MICE | 68 (22%) | 238 | 97 (32%)[b] | 209 | 15,360 | 2.0% |
| A. BCL-2 MICE | 38 (26%) | 109 | 50 (37%) | 87 | 7920 | 1.9% |

a. Canonical $V_H$ gene from codons 17–98.
b. Percentages of mutations clustered at mutational hot spots for each group are specified in parentheses.

The apparent contradictions of the observations between our results and those of Kuo et al., (1999) may be attributed to both differences in the nature of the transgenic mice and the antigen systems used for these studies. For example, the Eμ*bcl*-2–22 transgene is driven by the SV40 promoter and was present on the BALB/c background whereas the M23 Bcl-2 transgene is driven by the human Bcl-2 promoter and was present on the strain A background. The use of different promoters may result in different levels of expression at specific stages of B cell differentiation contributing to the conflicting outcomes. In addition, alteration of genetic background has been previously shown to influence the effects of enforced Bcl-2 expression on the development of autoimmunity (Strasser et al., 1992). Furthermore, the $V_H$S107 expressing clonotype that dominates the PC response in BALB/c mice does not appear to undergo affinity maturation, whereas canonical anti-Ars clonotypes in A/J mice clearly do. Therefore, most somatically mutated forms of the $V_H$S107 expressing clonotype, including dual reactive cells, may lack the affinity requisite for selection into the memory B cell compartment.

Although dual reactive hybridomas were easily isolated from Ars-immunized A. Bcl-2 mice, there were no significant elevations of serum anti-DNA antibodies in these mice. In this case our results are consistent with those of the anti-PC response in Eμ*bcl*-2–22 transgenic BALB/c mice, which reported that titers of serum anti-DNA antibodies in these mice did not rise following primary or secondary PC immunizations (Kuo at al., 1990). Erikson et al., (1991) have also reported that in mice transgenic for the 3H9 heavy chain, transgene expressing B cells with a low affinity for DNA are resident in the periphery and can be isolated as hybridomas, but produce undetectable levels of serum antibody. Further analysis of the same transgene expressing B cells, demonstrated that they were phenotypically distinct from non-transgenic B cells and that they were functionally compromised (Nguyen et al., 1997).

Hence, the inability of dual reactive cells in A. Bcl-2 mice to secrete antibody may be a consequence of a defect or defects in Ig-mediated signaling due to chronic autoantigen engagement. Reduced surface levels of co-stimulatory receptors, which play a role in modulating the response through the Ig receptor, may alter the signaling threshold of such cells when stimulated through membrane Ig. Alternatively, sIg-mediated signaling may be defective, as demonstrated using other autoreactive Ig transgenic systems (Cooke et al., 1994). Particularly, it has been demonstrated that anergic B cells exhibit a proximal block in the sIg signaling pathway which prevents activation of receptor-associated tyrosine kinases in response to soluble antigen (Cooke at al., 1994). Whether dual reactive B cells in A.Bcl-2 mice are anergic due to a B cell receptor signaling defect will require further study.

The anti-Ars serum antibodies that were elicited in A.Bcl-2 mice also exhibited evidence of normal affinity maturation. This is in accordance with earlier studies of the (4-hydroxy-3-nitrophenyl)acetyl (NP) response in Eμbcl-2–22 C57Bl/6 transgenic mice, which demonstrated that affinity maturation to the hapten NP was not affected by over-expression of Bcl-2 (Smith et al., 1994). In contrast, studies of the NP response in Bcl-$x_L$ transgenic mice have shown that over-expression of Bcl-$x_L$ influences affinity maturation (Takahashi et al., 1999). In these studies it was observed that low affinity anti-NP clonotypes predominantly participated in the anti-NP response of Bcl-$x_L$ mice whereas these clonotypes were minor participants in the NP response of control mice.

The differences in the observed effect of enforced Bcl-2 and Bcl-$x_L$expression may be attributed to the unique functions of these two different anti-apoptotic proteins. Although the proteins are members of the same family, they appear to function independently of one another and at different stages of B cell development. For example, GC B cells have been demonstrated to down regulate Bcl-2 expression while Bcl-$x_L$ has been shown to be expressed in the centrocyte subset of these B cells (Tuscano et al., 1996). If positive and negative selection of antibody V region mutants take place at distinct stages of the GC reaction, these processes may be differentially altered by enforced or over-expression of regulatory factors that normally act at specific stages of this reaction.

A detailed analysis of the somatic mutations present in the $V_H$genes expressed by a panel of hybridomas isolated from the anamnestic response of A. Bcl-2 mice revealed clustering of mutations to mutational hot spots at a frequency similar to controls. In contrast, studies from Kuo et al., (1997) using Eμbcl-2–22 transgenic BALB/c mice and the anti-PC GC B cell response, suggested that this normal hot spot clustering was perturbed by enforced Bcl-2 expression. As mentioned above, differences in the nature of the transgenic mice and antigen systems utilized may influence experimental outcomes of this type. In addition, Kuo at al., (1997) sampled the V genes expressed by GC B cells whereas our studies utilized hybridomas derived from anamnestic responses. We are currently directly examining the nature of somatic mutations introduced into canonical V regions during the anti-Ars primary GC response of A.Bcl-2 mice to determine if differences in hot spot clustering are apparent at this stage of B cell differentiation. Irrespective of the results of this study, our current data strongly suggest that B cells that are selected into the memory compartment undergo unaltered V region hypermutation in A.Bcl-2 transgenic mice. Therefore, alteration of the hypermutation process is unlikely to account for the dramatically increased frequency of dual reactive B cells in the memory compartment of these mice.

In total, our observations suggest that over-expression of Bcl-2 in the B cell compartment does not alter most aspects of a T cell dependent B cell response, including somatic hypermutation, serum antibody affinity maturation, and positive selection of high affinity B cells into the memory compartment. However, over-expression of Bcl-2 does perturb negative selection, ultimately allowing dual reactive B cells to enter this compartment. Thus, our studies suggest that positive and negative selection are two independent mechanisms working in concert to ensure the formation of a memory B cell population composed of B cells with an increased specificity for foreign antigen.

### MATERIALS AND METHODS

### Serology and mAb Binding Assays

Mice were immunized with 100μg Ars (p-azophenylarsonate)-KLH emulsified in CFA for the induction of primary responses and subsequently boosted with 50μg Ars-KLH in PBS for secondary and tertiary responses. Mice were bled via the retro-orbital sinus and Ars and DNA reactivities were analyzed by ELISA as described (Hande et al., 1998). Antibodies were elaborated using peroxidase-conjugated goat anti-mouse IgG (Jackson Immunoresearch, Inc. West Grove, PA) followed by o-phenylenediamine (Sigma Chemical Co. St. Louis MO). Affinity maturation of serum antibodies was determined by an altered ligand density ELISA, using two different coupling ratios of Ars to BSA. The bound antibodies were elaborated with a rat anti-mouse kappa antibody followed by Streptavidin-alkaline phosphatase (Southern Biotechnology Associates, Birmingham, AL).

### Immunohistochemistry

Spleens were removed, flash frozen, and sectioned as previously described (Hande et al., 1998). Frozen sections were thawed, rehydrated and treated as before. To determine Bcl-2 transgene expression, sections were incubated with a mouse anti-human Bcl-2 antibody (Genosys Biotechnologies, The Woodlands, TX), which was then detected with biotinylated goat F(ab')2 anti-mouse IgG (Southern Biotechnology Associates, Inc. Birmingham, AL). The slides were incubated with the antibodies for one hour. The slides were washed in TBS-BSA and then incubated with Streptavidin-Alkaline Phosphatase (AP) (Southern Biotechnology Associates, Inc. Birmingham, AL) for one hour. Germinal centers were identified with peanut agglutinin (PNA) coupled to horseradish peroxidase (HRP) (E-Y Laboratories, San Mateo, CA). Bound AP and HRP activities were visualized using Napthol AS-MX/Fast Blue BB Base and 3-aminoethylcarbazole, respectively.

### Determination of mAb Affinity

The affinities of purified Ars mAbs were determined by fluorescence quenching using Ars-N-acetyl-L-tyrosine as described before (Manser 1989). Ars affinities were calculated using a curve-fitting program provided by Jackie Sharon (Boston University School of Medicine, Boston, MA) as described previously (Rothstein and Gefter, 1983).

### Reverse Transcriptase-PCR and Nucleotide Sequencing

Total RNA was made from hybridomas as previously described (Manser, 1989) and then subjected to reverse transcriptase-PCR. $V_H$ and $V_\kappa$ PCR amplification was done using primers described previously (Hande et al., 1998). The RT-PCR products were purified from agarose gels using the Qiaex II Gel Extraction Kit (Qiagen, Chatsworth, CA) according to the manufacturer's instructions and were sequenced using the "fmol" sequencing kit (Promega Biotech Corp, Madison, WI).

### *Acknowledgements*

We thank Stanley Korsmeyer for the M23 Bcl-2 transgenic mouse, Jan Erikson for the anti-DNA ELISA protocol and all the members of the Manser laboratory for their many indirect contributions to this work. This work was funded by a grant from the NIH (AI38965).

### *References*

Berek C., and Milstein C. (1987). Mutational drift and repertoire shift in the maturation of the immune response. Immunol. Rev. **96**:23–41.

Betz A.G., Neuberger M.S., and Milstein C. (1993). Discriminating intrinsic and antigen-selected mutational hotspots in immunoglobulin V genes. Immunol. Today **14**:405–411.

Casson L., and Manser, T. (1995). Random mutagenesis of two CDR amino acids yields an unexpectedly high frequency of antibodies with increase affinity for both cognate antigen and autoantigen. J. Exp. Med. **182**:743–750.

Cooke M.P., Heath A.W., Shokat K.M., Zeng Y., Finkelman F.D., Linsley P.S., Howard M., and Goodnow C.C. (1994) Immunoglobulin signal transduction guides the specificity of B cell-T cell interactions and is blocked in tolerant self-reactive B cells. J. Exp. Med. **179**:425–438.

Erikson J., Radic M.Z., Camper S.A., Hardy R.R., Carmack C., and Weigert M. (1991). Expression of anti-DNA immunoglobulin transgenes in non-autoimmune mice. Nature **349**:331–334.

Han S., Zheng B., Dal Porto J., and Kelsoe G. (1995). In situ studies of the primary response to (4-hydroxy-3-nitrophenyl)acetyl. IV. affinity-dependent, antigen driven B cell apoptosis in germinal centers as a mechanism for maintaining self tolerance. J. Exp. Med. **182**:1635–1644.

Hande S., and Manser T. (1997). Single amino acid substitutions are sufficient to generate or enhance the specificity of two forms of an anti-Ars antibody variable region for DNA. Mol. Immunol. **34**:1281–1290.

Hande S., Notidis E., and Manser T. (1998). Bcl-2 obstructs negative selection of autoreactive, hypermutated antibody V regions during memory B cell development. Immunity **8**:189–198.

Kuo P., Alban A., Gebhard D., and Diamond B. (1997). Overexpression of bcl-2 alters usage of mutational hot spots in germinal center B cells. Mol. Immunol. **14**:1011 1018.

Kuo P., Bynoe M., and Diamond B. (1999). Crossreactive B cells are present during a primary but not secondary response in BALB/c mice expressing a bcl-2 transgene. Mol. Immunol. **36**:471–479.

MacLennan I.C.M. (1994). Germinal centers. Annu. Rev. Immunol. **12**:117–139.

Manser T., Parhami-Seren B., Margolies M.N., and Gefter, M.L. (1987a). Somatically mutated forms of a major anti-p-azophenylarsonate antibody variable region with dramatically reduced affinity for p-azophenylarsonate: by-products of an antigen driven immune response? J. Exp. Med. **166**:1456–1463.

Manser T., Wysocki L.J., Margolies M.N., and Gefter M.L. (1987b). Evolution of antibody variable region structure during the immune response. Immunol. Rev. **96**:141–162.

Manser T. (1989). Evolution of antibody structure during the immune response: the differentiative potential of a single B-lymphocyte. J. Exp. Med. **170**:1211–1230.

Manser, T. (1991). Regulation, timing and mechanism of antibody V-gene somatic hypermutation: lessons from the arsonate system. In Somatic Hypermutation in V-regions, E.J. Steele, ed. (Boca Raton, Florida: CRC), pp. 42–54.

Martinez-Valdez H., Guret C., de Bouteiller O., Fugier I., Bancherau J., and Liu Y.-J. (1996). Human germinal center B cells express the apoptosis-inducing genes Fas, c-myc, $P^{53}$, and Bax but not the survival gene bcl-2. J. Exp. Med. **183**:971–977.

McDonnell T.J., Deane N., Platt F.M., Nunez G., Jaeger U., McKeran J.P., and Korsmeyer S.J. (1989). bcl-2-immunoglobulin transgenic mice demonstrate extended B cell survival and follicular lymphoproliferation. Cell **57**:79–88.

Naparstek Y., Andre-Schwartz J., Manser T., Wysocki L.J., Breitman L., Stollar B.D., Gefter M.L., and Schwartz R.S. (1986). A single germline $V_H$ gene segment of normal A/J mice encodes autoantibodies characteristic of systemic lupus erythematosus. J. Exp. Med. **164**:614–626.

Nguyen K.-A. T., Mandik L., Bui A., Kavaler J., Norvell A., Monroe J.G., Roark J.H., and Erikson J. (1997). Characterization of anti-single-stranded DNA B cells in a non-autoimmune background. J. Immunol. **159**:2633–2644.

Pulendran B., Kannourakis G., Nouri S., Smith K.G.C., and Nossal G.J.V. (1995). Soluble antigen can cause enhanced apoptosis of germinal-center B cells. Nature **375**:331–334.

Rathbun G., Sanz I., Meek K., Tucker P., and Capra J.D. (1989). The molecular genetics of the arsonate idiotypic system. Adv. Immunol. **42**:95–125.

Rogozin I.B., and Kolchanov N.A. (1992). Somatic hypermutagenesis in immunoglobulin genes. II. influence of neighboring base sequences on mutagenesis. Biochimica et Biophysica Acta **1171**:11–18.

Rothstein T.L., and Gefter M.L. (1983). Affinity analysis of idiotype positive and idiotype negative Ars binding hybridoma proteins and Ars immune sera. Mol. Immunol. **20**:161–168.

Shapiro G.S., Aviszus K., Ikle D., and Wysocki L.J. (1999). Predicting regional mutability in antibody V genes based solely on di- and trinucleotide sequence composition. J. Immunol. **163**:259–268.

Sharon J., Gefter M.L., Wysocki L.J., and Margolies M.N. (1989). Recurrent somatic mutations in mouse antibodies to p-azophenylarsonate increase affinity for hapten. J. Immunol. **142**:596–601.

Shlomchik M.J., Aucion A.H., Pisetsky D.S., and Weigert M.G. (1987). Structure and function of anti-DNA autoantibodies derived from a single autoimmune mouse. Proc. Natl. Acad. Sci. USA **84**:9150–9154.

Shlomchik M.J., Litwin S., and Weigert, M. (1989). The influence of somatic mutation on clonal expansion. Prog. Immunol. **7**:415–435.

Shokat K., and Goodnow C.C. (1995). Antigen-induced B-cell death and elimination during germinal-center immune responses. Nature **375**:334–338.

Smith K.G.C., Weiss U., Rajewsky K., Nossal G.J.V., and Tarlington D. (1994). Bcl-2 increases memory B cell recruitment but does not perturb selection in germinal centers. Immunity **1**:803–813.

Smith D.S., Creadon G., Jena P.K., Portanova J.P., Kotzin B.L., and Wysocki L.J. (1996). Di- and tri-nucleotide target preferences of somatic mutagenesis in normal and autoreactive B cells. J. Immunol. **156**:2642–2652.

Strasser A., Harris A.W., and Cory S. (1992). The role of bcl-2 in lymphoid differentiation and neoplastic transformation. Current Topics in Microbiology and Immunology **182**:299–302.

Takahashi Y., Cerasoli D.M., Dal Porto J.M., Shimoda M., Fruend R., Fang W., Telander D.G., Malvey E.N., Mueller D.L., Behrens T.W., and Kelsoe G. (1999). Relaxed negative selection in germinal centers and impaired affinity maturation in bcl-$x_L$ transgenic mice. J. Exp. Med. **190**:399–409.

Tsiagbe V.K., Inghirami G., and G.S. Thorbecke (1996). The physiology of germinal centers. Critical Reviews in Immunology **16**:381–421.

Tuscano J.M., Druey K.M., Riva A., Pena J., Thompson C.B., and Kehrl J.H. (1996). Bcl-x rather than Bcl-2 mediates CD40-dependent centrocyte survival in the germinal center. Blood **88**:1359–1364.

Vora K., and Manser T. (1995). Altering the antibody repertoire via transgene homologous recombination: evidence for global and clone-autonomous regulation of antigen-driven B cell differentiation. J. Exp. Med. **181**:271–281.

Weiss U., and Rajewsky K. (1990) The repertoire of somatic antibody mutants accumulating in the memory compartment after primary immunization is restricted through affinity maturation and mirrors that expressed in the secondary response. J. Exp. Med. **172**:1681–1689.

Wysocki L.J., Gridley T., Huang S., Grandea A.G., and Gefter M.L. (1987). Single germline $V_H$ and $V_\kappa$ genes encoded predominating antibody variable regions elicited in strain A mice by immunization with p-azophenylarsonate. J. Exp. Med. **166**:1–18.

